# Fat for fuel: lipid metabolism in haematopoiesis

**DOI:** 10.1002/cti2.1098

**Published:** 2019-12-24

**Authors:** Gerard Pernes, Michelle C Flynn, Graeme I Lancaster, Andrew J Murphy

**Affiliations:** ^1^ Haematopoiesis and Leukocyte Biology Baker Heart and Diabetes Institute Melbourne VIC Australia; ^2^ Department of Immunology Monash University Melbourne VIC Australia

**Keywords:** cellular metabolism, haematopoiesis, lipid metabolism

## Abstract

The importance of metabolic regulation in the immune system has launched back into the limelight in recent years. Various metabolic pathways have been examined in the context of their contribution to maintaining immune cell homeostasis and function. Moreover, this regulation is also important in the immune cell precursors, where metabolism controls their maintenance and cell fate. This review will discuss lipid metabolism in the context of haematopoiesis, that is blood cell development. We specifically focus on nonoxidative lipid metabolism which encapsulates the synthesis and degradation of the major lipid classes such as phospholipids, sphingolipids and sterols. We will also discuss how these metabolic processes are affected by haematological malignancies such as leukaemia and lymphoma, which are known to have altered metabolism, and how these different pathways contribute to the pathology.

## Graphical abstract

Metabolic pathways are important regulators of haematopoiesis, where metabolism controls maintenance and cell fate. This review will discuss lipid metabolism in the context of haematopoiesis, that is blood cell development. We will also discuss how these metabolic processes are affected by haematological malignancies.

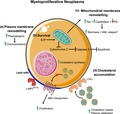

## Introduction

Blood cells are produced through a structured differentiation process occurring in the bone marrow (BM) termed haematopoiesis. Haematopoiesis begins when a haematopoietic stem cell (HSC) is subjected to specific intrinsic and extrinsic cues that promote proliferation and differentiation that gives rise to leucocytes, erythrocytes and platelets. Haematopoiesis is an ongoing process, required for the constant repopulation of blood over the life of an organism.^1^ The factors driving this process and the subsequent lineage biasing and commitment phases have been well studied, with the underlying transcriptional changes attracting the most attention.

In recent years, HSC energy metabolism has emerged as an important regulator of cell fate, with energy requirements dictating whether a HSC differentiates or remains quiescent.^2^ Importantly, lipid metabolism, primarily fatty acid oxidation (FAO), is utilised by both the most primitive HSCs and more committed progenitor populations to aid in self‐renewal and differentiation.^3^, ^4^ While FAO is a crucial aspect of HSC biology, lipid metabolism itself is a broader entity. The global lipid landscape, that is the cellular lipidome, is composed of a theoretical ~100 000 lipids.^5^ As this diversity implies, lipids are far more than just cellular fuel sources; they provide structural integrity to cells by forming membrane bilayers and can further serve as signalling intermediates.^6^

Our understanding of the interplay between haematopoiesis and lipid metabolism has primarily focused on the contribution of FAO to energy metabolism. Compared to this, how the nonoxidative lipid metabolic pathways contribute to HSC function has remained out of the limelight. However, studies are now beginning to address this topic in more detail. In this review, we discuss various pathways involved in lipid metabolism and how they pertain to haematopoiesis. Further, we discuss how these pathways are altered and their contribution in haematological malignancies such as leukaemia.

## Haematopoiesis

Mature blood cells are formed via a number of developmental lineages through the progressive proliferation and differentiation of stem and progenitor cells in a process called haematopoiesis.^7^ At the apex point of haematopoiesis sits the HSC which is the common progenitor for all blood cells. In its most primitive and quiescent state, the HSC is referred to as a long‐term HSC (LT‐HSC) and relies primarily upon anaerobic glycolysis for energy.^8^ Upon cellular stress, these LT‐HSCs progressively proliferate and differentiate through to short‐term HSCs (ST‐HSCs) and subsequent multipotent progenitor cells (MPPs), which increase their rate of proliferation while progressively losing their capacity for self‐renewal.^9^ Through this process, these progenitors also begin to develop a bias towards the production of specific blood cell lineages. This consequently results in the differentiation of these MPPs into the lineage‐committed progenitors: the common lymphocyte progenitor (CLP)^10^ and the common myeloid progenitor (CMP).^11^ Importantly, while there is consensus upon the concept of lineage bias and commitment through haematopoiesis, our understanding of the precise definition of lineage‐committed progenitors and their resulting mature blood cell populations continues to evolve. Traditionally, the haematopoietic hierarchy involves the differentiation of the CMP into megakaryocyte–erythroid progenitors (MEPs) and granulocyte–macrophage progenitors (GMPs), while the CLPs further differentiate to produce B cells, T cells and NK cells.^7^ While some studies suggest that alternative pathways of lineage commitment may exist – including the identification of lymphoid‐primed multipotent progenitors (LMPPs)^12^ that are proposed to differentiate either into CLPs or directly into GMPs as well as CMP‐ and/or MEP‐independent platelet production – the majority of lineage tracing studies support the traditional model of haematopoiesis with minor variations.^13^ The regulation of haematopoiesis across the different stages of differentiation and commitment is crucial to modulating the balance of the mature blood cell compartments. Haematopoiesis is intrinsically regulated by stem and progenitor cell metabolism, with different metabolic parameters required for both proliferation (through symmetric mitosis) and differentiation (through asymmetric mitosis) across the various progenitors.^14^ Haematopoiesis is thus modulated by factors that influence cellular metabolism, including inflammatory signals and nutrient availability.

## Lipid metabolism in haematopoiesis

**Figure 1:**
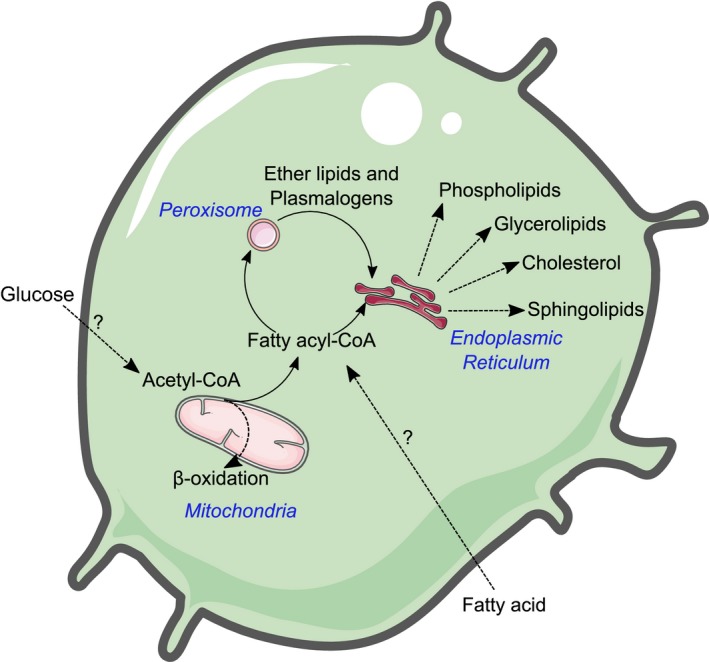
A brief overview of lipid metabolism in immune cells. Acetyl‐CoA can be derived either from glycolysis or from extracellular FAs that can be used to fuel B‐oxidation or can be converted into fatty acyl‐CoAs, committing them to nonoxidative lipid metabolism. Fatty acyl‐CoAs are further processed in the endoplasmic reticulum where they produce lipids such as phospholipids, glycerolipids, sterols and sphingolipids. Fatty acyl‐CoAs can also be redirected to the peroxisome where they are converted to fatty alcohols, the rate‐limiting step in plasmalogen biosynthesis where they finish processing in the endoplasmic reticulum.

**Figure 2:**
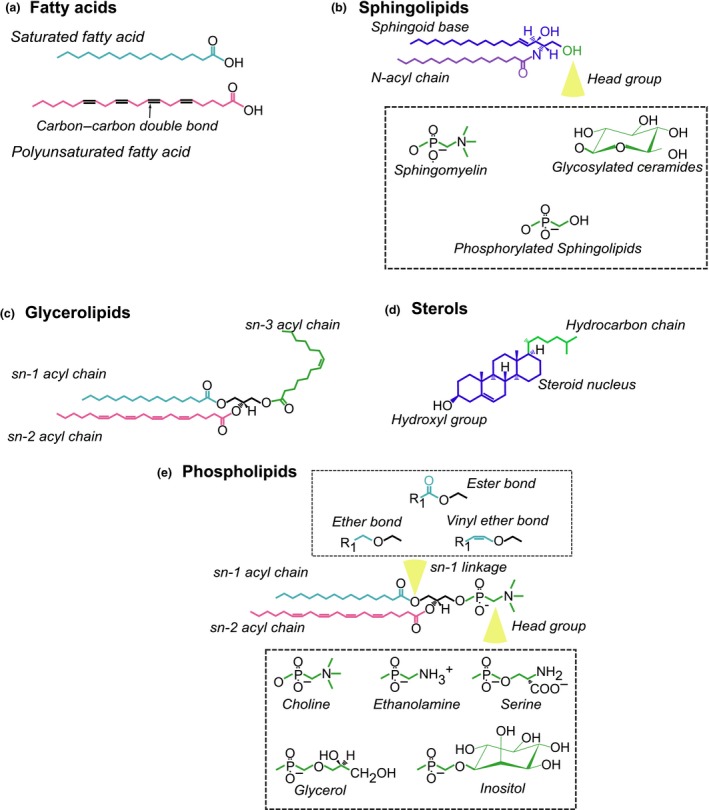
Representative chemical structures of the main lipid classes in eukaryotic cells. **(a)** Fatty acids: Consisting of a hydrocarbon chain connected to a carboxyl group. The hydrocarbon chain can either be saturated or be unsaturated, indicated by the presence of a carbon–carbon double bond (unsaturated). **(b)** Sphingolipids: An acyl chain is connected to a sphingoid base (typically sphinganine) to produce ceramide. Through further metabolic reactions, the head group region can change to yield complex sphingolipids such as sphingomyelin and glycosylated ceramides. **(c)** Glycerolipids: Fatty acids can be attached at the *sn‐1, sn‐2* and *sn‐3* positions of the glycerol backbone to produce monoacylglycerol (MAG) [1 fatty acid], DAG [2 fatty acids] and TAG [3 fatty acids]. **(d)** Sterols: An isoprenoid‐based lipid with four hydrocarbon rings where a hydroxyl group located on one end and a hydrocarbon chain attached at another. **(e)** Phospholipids: Two fatty acids connected to a glycerol backbone at the *sn‐1* and *sn‐2* position. At the *sn‐3* position is a variable head group which produces the major phospholipid species in eukaryotes. Further, the *sn‐1* linkage defines can alter the phospholipid species.

The role of oxidative lipid metabolism is well established in haematopoiesis. While free FAs (FFAs) are used as an energy source by both HSCs and progenitor cells (reviewed elsewhere^4^), lipid metabolism is much broader, constituting a variety of metabolic pathways responsible for producing sphingolipids, glycerolipids, phospholipids and sterols (Figures 1 and 2).^15^ Moreover, these metabolic pathways can influence one another, that is perturbations in one metabolic pathway can also affect another.^16^ These lipids have a vast array of functions including energy stores, structural components for membranes and signalling intermediates. In the following sections, the major lipid classes and their associated metabolic pathways will be discussed pertaining to their roles in haematopoiesis, these are also summarised in Table 1.

### Sphingolipids

Sphingolipids represent a group of lipids with roles in membrane integrity and cellular signalling, all characterised by the presence of a sphingoid backbone (Figure 2b). At the centre of sphingolipid metabolism is the bioactive intermediate ceramide, which consists of an acyl chain connected to the sphingoid base and is either produced through *de novo* synthesis or the catabolism of complex sphingolipids.^17^ Through multiple enzymatic reactions, ceramide can be metabolised into membrane constituents, such as sphingomyelin, ganglioside and cerebrosides, or into signalling molecules including ceramide‐1‐phosphate (C1P) or sphingosine‐1‐phosphate (S1P).^18^ Ceramides also have important roles as a signalling intermediate in numerous cells.

**Figure 3:**
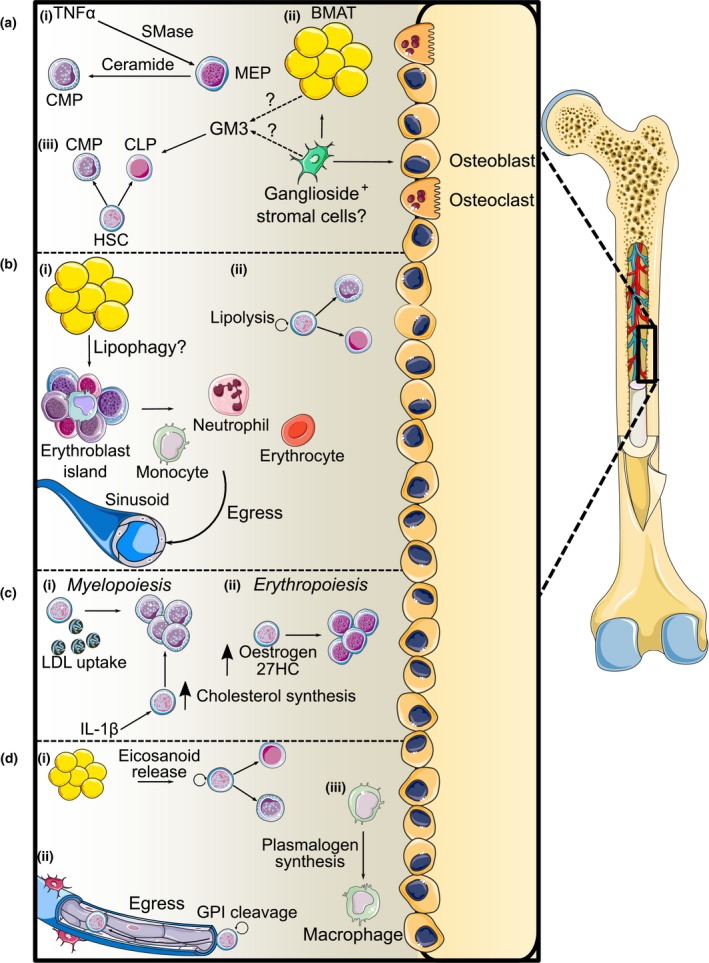
Nonoxidative lipid metabolism in haematopoiesis. **(a)** Sphingolipids: **(i)** TNF‐α activates sphingomyelin hydrolysis through sphingomyelinase (SMase), changing erythroid progenitors towards a myeloid phenotype. **(ii)** Ganglioside‐expressing stromal cells (presumably a subset of the leptin receptor^+^ stromal cell population) differentiate into adipocytes and osteoblasts. **(iii)** Stromal cell‐derived gangliosides support both myelopoiesis and lymphopoiesis directly or by supporting the BM niche. **(b)** Glycerolipids: **(i)** Budding lipid droplets from BMAT interact with erythroblast island macrophages, supporting myelopoiesis and erythropoiesis and the egress of their mature cells from the BM. **(ii)** HSCs utilise lipolysis through LPL and LAL to regulate HSC differentiation. **(c)** Cholesterol: **(i)** Increasing cholesterol levels through LDL uptake and inflammatory signalling‐induced cholesterol biosynthesis promotes myelopoiesis. **(ii)** Oestrogen and 27HC‐induced activation of the oestrogen receptor promotes erythropoiesis. **(d)** Phospholipids: **(i)** Phospholipid catabolism liberates polyunsaturated FAs for eicosanoid synthesis which maintains HSC self‐renewal and regulates myelopoiesis and erythropoiesis. **(ii)** PLCβ2‐mediated GPI cleavage induces HSPC egress from the BM. **(iii)** Plasmalogen synthesis is observed during monocyte‐to‐macrophage differentiation.

In respect to cell fate, the ceramide biosynthetic pathway has been shown to be important following tumor necrosis factor‐α (TNF‐α)‐induced sphingomyelin hydrolysis, where the recycling of sphingomyelin into ceramide determines whether a cell undergoes erythropoiesis or myelopoiesis (Figure 3ai).^19^ Sphingomyelin‐derived long‐chain ceramides were increased upon TNF‐α treatment of erythroid‐primed CD34^+^ haematopoietic stem and progenitor cells (HSPCs), which was concomitantly associated with a morphological shift resembling myeloid progenitor cells and an increase in the transcription factors PU.1, GATA1 and GATA2 along with inhibition of autophagy (Table 1). Interestingly, it was noted that S1P treatment abrogated myelopoiesis and instead promoted erythropoiesis, presumably through its antagonistic effects on ceramide.

Sphingosine‐1‐phosphate itself has chemoattractant properties on HSCs. The Obinata group have shown that S1P induces HSPC invasion into stromal cell layers^20^ in an S1P receptor 1 (S1PR1)‐dependent manner.^21^ Interestingly, S1P is released from erythrocytes, the major S1P reservoir in peripheral blood, in response to treatment with a mobilising agent such as granulocyte colony‐stimulating factor (G‐CSF) or zymosan to promote HSPC mobilisation in a C‐X‐C motif chemokine 12 (CXCL12)‐independent manner.^22^ This study also demonstrates that previous exposure to S1P inhibits HSPC mobilisation when re‐exposed to S1P which could be explained through two mechanisms: (1) the receptor S1PR1 is downregulated when exposed to high concentrations of S1P in circulation, and (2) S1P binds to S1PR2, another S1P receptor with chemotactic‐inhibitory effects.^22^, ^23^ Conversely, mice with a sphingosine kinase 1 (Sphk1) deletion had defective reconstitution following transplantation, suggesting that S1P production is required for HSPC homing to the BM.^24^ S1P has also been demonstrated to induce CXCL12 secretion from mesenchymal stem cell populations in the BM niche, promoting the egress of progenitor cells in a reactive oxygen species (ROS)‐dependent manner.^25^

Other complex sphingolipids have also been shown to modulate haematopoiesis. Gangliosides, glycosylated ceramides linked to one or more sialic acid moieties, have been demonstrated to both induce and prevent myeloid differentiation *in vitro* (Figure 3a). Kaucic *et al*. noted that both brain‐derived and tumor‐derived complex gangliosides such as GD1a, GD1b and GT1 inhibited myelopoiesis.^26^ Multiple groups have validated these findings, showing that numerous tumor‐derived ganglioside species inhibit myeloid differentiation and promote apoptosis.^27^, ^28^, ^29^ Unlike sphingomyelin, gangliosides and their upstream metabolites, glycosylated ceramides, are derived from the BM microenvironment or niche, suggesting that haematopoietic progenitor cells are incapable of synthesising these lipids.^30^ Studies from the Borojevic and Guma laboratories demonstrate that stromal‐derived gangliosides, specifically GM1 and GM3^31^ and GD1a,^32^ are incorporated into myeloid‐like cells to support their differentiation and that inhibiting ganglioside production negatively impacted cell survival and proliferation, suggesting a supportive role for gangliosides in myelopoiesis (Figure 3aii). Whether these cells intrinsically produce these sphingolipids has yet to be determined. The importance of these stromal‐derived lipids has been demonstrated in both myelopoiesis and lymphopoiesis *in vitro* and *in vivo*. BM stromal cells express gangliosides in both humans and mice.^33^, ^34^, ^35^ Xu *et al*. have shown that ganglioside‐expressing murine stromal cells exhibit a stronger proliferative phenotype.^34^ These cells differentiate more readily into adipocytes and osteoblasts, cellular components of the BM niche that support haematopoiesis and are likely leptin receptor^+^ mesenchymal stromal cells or a subset thereof (Figure 3aii). Further, the transport of GM3 into myeloid progenitors has been suggested to sustain lipid raft formation, allowing for enhanced granulocyte–macrophage (GM)‐CSF signalling, thus enhancing myeloid proliferation.^30^ While these findings have been performed in *in vitro* cultures, it is yet to be determined whether BM stromal cells transport these complex sphingolipids to myeloid progenitors *in vivo*. However, the Frenette group have demonstrated that glycosylated ceramides are crucial in lymphopoiesis (Figure 3aiii).^36^ Lymphoid organs (lymph nodes, spleen and thymus) from UDP‐galactose:ceramide galactosyltransferase (*Cgt*)‐null mice were atrophied because of a decrease in cell number from the resultant arrested B‐/T‐cell development due to the inability of stromal cells to form fibronectin‐rich niches in the BM to support their development.^36^

Together, sphingolipids influence haematopoietic lineage commitment in both a cell‐autonomous manner and through maintaining the BM niche (Table 1). Further research is needed to understand whether stromal‐derived sphingolipids interact with haematopoietic cells, thus directly supporting haematopoiesis and lineage commitment.

### Glycerolipids

Glycerolipids consist of one, two or three FAs connected to a glycerol backbone (Figure 2c). Primarily, intracellular glycerolipids are found as triacylglycerol (TAG), stored in neutral lipid depots known as lipid droplets where they act as energy reservoirs. Diacylglycerol (DAG) is an important lipid intermediate involved in many signalling processes.^37^ Within the BM niche, BM adipose tissue (BMAT) has been shown to influence haematopoiesis in addition to its canonical roles as an endocrine tissue and energy store.^38^

Direct interactions between BMAT and haematopoietic cells have recently been visualised by the Scheller group, where they applied focused ion beam scanning electron microscopy to examine how these cells interact within the BM niche.^38^ This study showed that budding lipid droplets from BMAT interacted with maturing myeloid cells and phagocytic reticular cells or erythroblast island macrophages in the BM, where they may act to support myeloid and erythroid maturation and aid in their release into sinusoids (Figure 3bi). Furthermore, these reticular phagocytes were shown to have an extensive network of lipid droplets that associated with nucleated erythroblasts and, to a lesser extent, granulocytes. Evidence from their work suggests that BMAT is spatially positioned to affect both myelopoiesis and erythropoiesis, potentially serving as an energy reservoir to fuel their differentiation.

Fatty acid utilisation, in particular FAO, is crucial for HSC maintenance. The Pandolfi group demonstrated that the loss of peroxisome proliferator‐activated receptor (PPAR)‐δ‐mediated mitochondrial FAO impacted long‐term reconstitution capacity by inducing differentiation and a loss of asymmetric division.^39^ This FAO was further shown to regulate mitophagy as a means to regulate HSC expansion.^40^ Other variations of autophagy also aid in HSC self‐renewal, contributing to other processes besides FAO.^3^ Lipophagy is the catabolism of lipid droplets to liberate FAs and other lipid molecules during starvation or nutrient stress (Figure 3bii).^41^ The enzymatic machinery involved in lipid catabolism such as lipoprotein lipase (LPL)^42^, ^43^ influences HSC biology. Liu *et al*. have shown in both mice and zebrafish that LPL activity is crucial for definitive haematopoiesis, liberating the polyunsaturated FA (PUFA) docosahexaenoic acid (DHA, 22:6) from TAG pools.^43^ Deleting *apoc2*, the gene encoding for an obligatory cofactor for LPL activity, attenuated the expression of *Runx1*, *Cmyb*, *Beta‐globin* and *Rag1*, indicating erythropoietic and lymphopoietic defects alongside with hyperlipidaemia. Interestingly, their studies in *Apoc2^−/−^* mice showed that the anaemic phenotype was alleviated 4 months after weaning; however, these animals still displayed a decreased leucocyte count, driven mainly by lower myeloid cell counts. Chang *et al*. have also noted decreased myeloid cellularity in *Lpl^−^* ^/^ *^−^* mice, which was concomitant with a decreased expression of key myeloid transcription factors as well as G‐, GM‐ and macrophage (M‐)CSF and their associated receptors.^42^ They attributed this deficiency to PPAR‐γ activation and a concomitant decrease in M‐CSF production and, interestingly, a loss of LPL‐CSF‐R interaction at the membrane, where LPL facilitates M‐CSF binding to its cognate receptor.

Contrastingly, work from Du and colleagues implicates lysosomal acid lipase (LAL) as an important regulator of haematopoiesis. LAL‐deficient animals were noted to have neutrophilia in the lungs^44^ attributed to the upregulation of anti‐apoptotic proteins, specifically Api6, and an increase in macrophage proliferation and foam cell formation. This also affected myeloid progenitors where *Lal^−^* ^/^ *^−^* mice had increased levels of HSPCs, CMPs and GMPs that led to an increase in myeloid cells.^45^ LAL is also required for normal T‐cell development in the spleen and thymus as *Lal^−/−^* mice exhibit excessive neutral lipid deposition and macrophage accumulation in these organs. *Lal* depletion impairs T‐cell maturation in the thymus and results in an increase in splenic T‐cell apoptosis as well as impairing proliferation and cytokine production in response to activation both *in vivo* and *in vitro*.^46^

Lipolysis is just one route whereby cells derive FAs. They can also be synthesised *de novo* or taken up from the external environment.^47^ Recent evidence from memory T cells suggests that long‐lived cells utilise both FAO and *de novo* synthesis for survival.^48^, ^49^ While having both pathways active simultaneously is bioenergetically wasteful, this may have an effect on HSC survival. If HSCs were to utilise both glycolysis and FAO in parallel,^8^, ^40^, ^50^ this may allow HSCs to maintain their quiescent nature via glycolysis, while small amounts of FAO could be used to maintain mitochondrial function.

Taken together, there is an emerging literature indicating an important role for neutral lipid storage and turnover in HSPC differentiation (Table 1). While FAO is used by HSCs to maintain their self‐renewal capacity, it would be of interest to observe FA flux during the different stages of haematopoiesis and how this is regulated.

### Sterols

Sterols are found within lipid bilayers where they maintain membrane fluidity and lipid raft formation. In mammals, these properties are regulated by cholesterol (Figure 2d). Cholesterol is produced *de novo* from acetyl‐CoA incorporation into the mevalonate pathway. The downstream metabolite of the mevalonate pathway, squalene, can be utilised by the Kandutsch–Russell and Bloch pathways to produce cholesterol.^51^

Haematopoietic stem and progenitor cell mobilisation has been observed under hypercholesterolaemic conditions such as atherosclerosis where increased myelopoiesis occurs in the BM and in secondary sites like the spleen to produce monocytes and neutrophils that infiltrate the plaque due to defects in cholesterol efflux^52^ (Figure 3ci). This myeloproliferative phenotype has been mechanistically demonstrated by our laboratory and others to be a consequence of membrane cholesterol accumulation and subsequent lipid raft formation where the common β subunit of the IL‐3/GM‐CSF receptor or CD131 becomes more highly expressed.^53^, ^54^, ^55^, ^56^ Moreover, hypercholesterolaemia promotes HSPC mobilisation by increasing plasma CXCL12 levels and disrupting the CXCL12/CXCR4 axis (Table 1).^57^ Activating the transcription factor, liver X receptor (LXR) prevents the myeloproliferative phenotype by activating cholesterol efflux pathways.^58^

While the link between cholesterol transport and homeostasis and haematopoiesis is well defined, much less is known regarding how cholesterol synthesis and degradation influence this process. Cholesterol metabolites have been demonstrated to influence haematopoiesis through their effects on differentiation and HSPC mobilisation. Nakada *et al*. demonstrate that oestrogens regulate the rate of HSC division, with female mice having more dividing HSCs compared to males, and oestradiol administration in both male and female mice increased HSC division.^59^ Oestradiol also induces HSC differentiation into MEPs as noted by their increased abundance in the BM and promotes extramedullary erythropoiesis (Figure 3cii). Interestingly, oestradiol does not induce HSPC mobilisation. Instead, this is induced by another cholesterol metabolite, 27‐hydroxycholesterol (27‐HC), through a G‐CSF‐independent manner but mediated in part by CXCL12. Notably, this 27‐HC‐induced haematopoiesis has been shown to be necessary for HSPC mobilisation and extramedullary haematopoiesis during pregnancy but not under any other haematopoietic insults.^60^

Cholesterol metabolism in HSCs is also affected during trained immunity, a phenomenon resembling immunological memory in myeloid cells denoted by epigenetic and metabolic reprogramming resulting in an enhanced response to a secondary infection.^61^, ^62^ Accumulation of the cholesterol precursor, mevalonate in β‐glucan‐ and oxidised low‐density lipoprotein (oxLDL)‐treated monocytes, activates IGF1 and mTOR signalling pathways concomitant with histone modifications.^63^ Stimulation with β‐glucan induces changes in the cytokine milieu, particularly IL‐1β (Figure 3ci), which effects haematopoietic progenitors in the BM leading to increased proliferation, which we previously noted in obesity.^64^ IL‐1β also induces an upregulation in cholesterol biosynthesis and transport as well as a decrease in cholesterol efflux.^65^ This same phenotype was observed by Christ *et al*. where myeloid progenitors from mice fed a western‐type diet exhibited the trained immunity phenotype mediated through IL‐1 signalling downstream of the NLRP3 inflammasome.^66^ Lowering cholesterol levels by blocking 3‐hydroxy‐3‐methylglutaryl (HMG)‐CoA reductase through statins reversed the enhanced myelopoiesis observed in β‐glucan treatment, presumably by preventing lipid raft formation and the expression of CD131, while also preventing cytokine production after restimulation.^63^, ^65^ A recent prospective cohort study demonstrated that monocytes derived from patients with familial hypercholesterolaemia displayed the trained immune phenotype compared to healthy controls.^67^ Interestingly, this effect was not ablated following 3 months of statin treatment.^68^ The use of statins to abolish this effect is complicated as blocking cholesterol synthesis in the HSPCs would likely also induce the expression of the low‐density lipoprotein receptor (LDLr), an important cholesterol uptake pathway in HSPCs, providing an alternative source of lipid for the cell (i.e. as seen in the liver).^68^

Cholesterol homeostasis is an important aspect of the metabolic regulation of haematopoiesis. Following extensive research regarding cholesterol transport and efflux out of the cell which demonstrated the importance of cholesterol loading in haematopoiesis, further research now indicates the significance of intracellular cholesterol metabolism and key cholesterol metabolites as important mediators of haematopoiesis.

### Phospholipids

Phospholipids are primary membrane constituents that consist of two acyl chains linked to a glycerol backbone with a phosphorylated head group. Depending on the variable head group, phospholipids can exist as either phosphatidylcholine (PC), phosphatidylethanolamine (PE), phosphatidylglycerol (PG), phosphatidylinositol (PI) or phosphatidylserine (PS; Figure 2e).^15^

The major membrane lipids PC and PE also serve as substrate reservoirs for polyunsaturated FA (PUFA) metabolism. PUFAs are cleaved from phospholipids in a phospholipase‐A2 (PLA_2_)‐dependent manner where they are then used to synthesise eicosanoids; a collective term for PUFA‐derived metabolites involved in signalling and includes prostaglandins, leukotrienes and special pro‐resolving mediators.^69^

Prostaglandin E_2_ (PGE_2_) synthesis, mediated by cyclooxygenase (COX) enzymes, increases HSC self‐renewal and their capacity to repopulate the BM and whole kidney marrow *ex vivo* in mice and zebrafish^70^ (Figure 3di). The Zon group have demonstrated that this primarily occurs via Wnt signalling by promoting protein kinase A (PKA)‐mediated β‐catenin degradation.^71^ The Pelus laboratory further elucidated the effects of PGE_2_ on haematopoietic cells by demonstrating that PGE_2_ signalling through the E‐prostanoid 4 receptor regulates HSPC egress from the BM. This was through both a cell‐autonomous effect in the HSCs where PGE_2_ acts directly to retain them in the BM, as well as cell‐extrinsic effects on the BM niche where whereas PGE_2_ decreases osteopontin levels in BM stromal cells (Table 1).^72^

While the COX enzymes are responsible for prostaglandin synthesis, eicosanoids are produced through two other main enzyme families, the lipoxygenases (LOX) and the cytochrome P450 epoxy hydrolases.^69^ Like PGE_2_, LOX‐derived eicosanoids, primarily hydroxyeicosatetraenoic acid (HETE), have also been shown by Kinder *et al*. to be required for HSC self‐renewal.^73^ 12/15‐LOX (Alox15)‐null mice had greater LT‐HSC proliferation and differentiation and also exhibited impaired capacity to repopulate the BM following transplantation. This was associated with dysregulated Wnt signalling and an accumulation of ROS.

**Table 1:** Summary of the various lipid species discussed and their roles in haematopoiesis

Lipid species	Effect on haematopoiesis
Ceramide	Increased ceramide synthesis via sphingomyelin hydrolysis influences myelopoiesis (increased PU.1, GATA1 and GATA2 expression)
Sphingosine‐1‐phosphate	Synthesis prevents the effects of ceramide presumably through its antagonistic effects or by decreasing ceramide availability HSPC chemoattractant
Glycosylated ceramides	Support lymphopoiesis by influencing stromal cell niche formation
Gangliosides	Regulate myelopoiesis via their potential incorporation into myeloid progenitor cells by promoting lipid raft formation Stromal cell populations enriched in gangliosides readily differentiate into adipocytes and osteoblasts supporting haematopoiesis within the BM niche
Glycerolipids	Glycerolipids within the BMAT bud off and support myelopoiesis and erythropoiesis as well as the egress from the BM Store FAs that can be used to provide substrate for differentiation and proliferation Diacylglycerol liberated from phospholipids via PLCβ2 has chemotactic effects
Cholesterol	Hypercholesterolaemia induces HSPC mobilisation due to increased lipid raft formation and the localisation of CD131 and increased CXCL12 production
Oestrogens	Regulate HSC division Promote erythropoiesis
27‐Hydroxycholesterol	Induces HSPC mobilisation independent of G‐CSF
Prostaglandin E_2_	Derived from membrane phospholipids Increases HSC self‐renewal, repopulation and egress Regulates HSC differentiation
Hydroxyeicosatetraenoic acid	Involved in BM repopulation and limiting LT‐HSC self‐renewal
Phospholipids	Serve as a reservoir for signalling lipids GPI cleavage via PLCβ2 allows HSC egress Cardiolipins needed for neutrophil development
Ether lipids	Involved in monocyte‐to‐macrophage differentiation Synthesis may be crucial for neutrophil development

Studies dating back to the 1970s have shown that PGE_2_ inhibited myeloid differentiation *in vitro*.^74^ The Moore group mechanistically probed this by demonstrating that monocyte‐ or macrophage‐derived PGE_2_ established a negative feedback loop to limit progenitor cell proliferation and differentiation in response to CSF‐induced monocytic differentiation.^75^ This suppressive effect was also demonstrated in lymphopoiesis by the Kincade group where B‐cell precursors were decreased following treatment with a PGE_2_ analogue, 16,16‐dimethyl PGE_2_.^76^ Moreover, CFUs from B‐cell precursors (CD45R^+^, sIgM^−^) enriched BM following PGE_2_ treatment had impaired colony formation in response to IL‐7 stimulation and increased apoptosis.^76^

Their findings also hint at this process being regulated by stromal cells, as cocultures with stromal cells reduced PGE_2_‐induced apoptosis, while stem cell factor (SCF), largely produced by stromal cells, reduced apoptosis and partially restored B‐cell progenitor levels. Interestingly, the same group also showed that adiponectin induces PGE_2_ release from stromal cultures and adiponectin dampens lymphopoiesis in the presence of stromal cells in a cyclooxygenase 2 (COX‐2)‐dependent manner, indicating that stromal‐derived PGE_2_ can also suppress lymphopoiesis.^77^ Interestingly, their results also indicate that adiponectin‐induced PGE_2_ production stimulates myelopoiesis as a consequence (Figure 3di).

While eicosanoids are produced from PLA‐mediated phospholipid cleavage, another lipase, PLC, also influences haematopoiesis. PLC plays roles in signal transduction by generating inositol triphosphate and DAG species. A haematopoietic‐specific isoform of PLC, PLCβ2, has been identified to be required for multiple chemoattractant‐induced responses including Ca^2+^ efflux and superoxide production.^78^ Despite this, PLCβ2 also promotes HSPC mobilisation by cleaving glycosylphosphatidylinositol (GPI) located in lipid rafts, thus disrupting this structure, resulting in decreased expression of CXCR4 at the membrane level and allowing HSPC egress from the BM (Figure 3dii).^79^

Defects in phospholipid metabolism may impact myelopoiesis. Patients with Barth syndrome, an X‐linked recessive disease caused by mutations in tafazzin (Taz), an enzyme involved in the production of the mitochondrial phospholipid cardiolipin,^80^ present with neutropenia. BM aspirates from these patients exhibit an arrested myeloid development at the myelocyte stage with upstream progenitors having abnormal mitochondrial structures.^81^ As HSCs derived from these patients are hard to obtain, Makaryan *et al.* demonstrate that Taz knockdown HL60 cells undergo apoptosis. This was not attributed to changes in cardiolipin levels,^82^ but to mitochondrial dysfunction with increased cytochrome c release and caspase 3 activation. The authors speculate that the reason no difference was observed in cardiolipin levels in these cells was that the transfection time was insufficient. It may also be possible that Taz knockdown alters the membrane composition through a different mechanism. While this study only assessed cardiolipin levels, Taz also acts on PC species to help produce cardiolipins by transferring an acyl chain from PC to cardiolipin.^80^ With cardiolipin being recently shown to regulate mitochondrial function by maintaining structural integrity,^83^ the potential disruption of the PC‐lysoPC ratio within the mitochondrial membrane may alter its integrity and, subsequently, its function.

Phospholipid catabolism is necessary for liberating substrates to produce bioactive lipids to regulate HSC function and fate (Table 1). While being reservoirs for lipid mediators, they are primarily major membrane constituents, shaping cells by providing the bulk of membrane bilayers. However, little is known about how the membrane itself is organised. This lipid pool is quite diverse and arises from variations made in the acyl chains at *sn‐1* and *sn‐2* positions and changes in the head group region. The membrane composition of the HSC may be well suited to allow for certain functions; however, this is yet to be determined and warrants investigation.

### Ether lipids and plasmalogens

Structurally similar to phospholipids, plasmalogens are also membrane constituents; however, they differ in their chemical structure where the *sn‐1* position contains an alkyl or alkenyl group instead of an acyl group. Ether lipid synthesis is mediated by a separate enzymatic pathway that largely takes place in the peroxisome.^84^ This chemical disparity also makes them functionally distinct from diacyl phospholipids, whereby the modified bond at the *sn‐1* position produces a thicker and more rigid membrane due to an increased packing density (Figure 2e).^84^ The distribution between diacyl phospholipids and ether lipids differs depending on the cell type; however, in leucocytes, they have a moderate to high abundance.^85^

The plasmalogen content increases upon myelopoiesis as evidenced in a study by the Schmitz group.^86^ Human monocytes when differentiated into macrophages increase their plasmalogen content alongside a change in FA preference with more saturated to lowly unsaturated FAs (1‐3 carbon–carbon double bonds) found in the plasmalogens, implicating lipid remodelling as a feature of macrophage development (Figure 3diii).^86^

Furthermore, Lodhi *et al*. have implicated plasmalogen metabolism as an important regulator in neutrophil physiology as mouse models deficient in peroxisomal reductase‐activating PPAR‐γ (*PexRAP*), a gene involved in the export of peroxisomal metabolites, exhibited neutropenia due to a loss of membrane integrity and subsequent ER stress‐induced apoptosis.^87^ However, their findings have been contested by Dorninger and colleagues who have shown that this neutropenia phenotype is not found in *Gnpat* knockout mice and in rhizomelic chondrodysplasia punctata (RCDP) who have a mutation in *Gnpat*, the first enzyme in ether lipid synthesis.^88^ Despite the apparent importance of plasmalogens in mature myeloid cells, it is unclear whether altered plasmalogen metabolism affects the upstream progenitors. Plasmalogens are also decreased as a consequence of trained immunity metabolic reprogramming after β‐glucan treatment, but interestingly was not accompanied by a compensatory increase in the levels of diacyl phospholipids. The regulatory mechanisms and functional consequences of this are currently not known, but this lipid remodelling may be necessary for the enhanced immune response seen upon trained immunity induction.

Collectively, plasmalogens appear to be important in myeloid cell development (Table 1). Whether these lipids can be broken down like their diacyl phospholipid counterparts to generate bioactive mediators has yet to be shown. Additionally, how this plasmalogen phenotype is programmed during haematopoiesis and its functional consequences remain to be examined.

## Lipid metabolism and leukaemia

**Figure 4:**
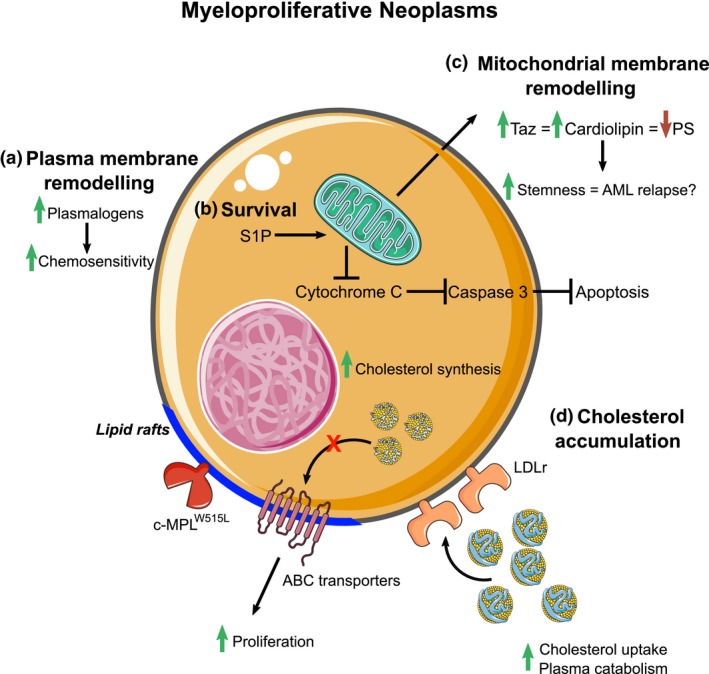
Lipid metabolism in myeloproliferative neoplasms. **(a)** Membrane remodelling at the plasma membrane and mitochondrial levels induces chemotherapeutic resistance and increases stemness. **(b)** Alterations in sphingolipid metabolism by shifting the metabolic flux towards S1P synthesis inhibit apoptosis. **(c)** Mitochondrial membrane remodelling occurs because of an increase in Taz activity leading to an increase in leukaemic stem cell stemness. **(d)** Increased cholesterol uptake and biosynthesis along with defective cholesterol efflux promote lipid raft formation, which houses raft‐dependent receptors such as c‐MPL enhancing cell proliferation.

The metabolic pathways that control the regulation of specific lipid classes are highly interconnected, and therefore, alterations, for example through gene deletion, within a given lipid metabolic pathway not only affect lipids within the manipulated pathway, but also have effects on the global lipid landscape more broadly.^16^ Accordingly, alterations in specific lipid metabolic pathways can have quite widespread cellular effects and can contribute to chronic diseases. Metabolic reprogramming is a hallmark of cancer with cancer cells and tumors exhibiting mass proliferation. For this to occur, they must generate the required biomass to expand.^89^ The first instance of metabolic reprogramming described in cancer is the switch to a glycolytic phenotype termed the Warburg effect.^89^ Furthermore, the lipid biosynthetic machinery is fuelled by the influx of carbon coming from both glucose and glutamine sources.^90^ Like other cancers, haematological malignancies such as leukaemia and lymphoma exhibit an aberrant lipid metabolic phenotype (Figure 4). As such, the global lipid landscape of cancer cell lines greatly differs from noncancerous cells.^91^ Indeed, recent evidence also shows that substantial lipid diversity exists within distinct types of cancer cells.^92^

Sphingolipid synthesis is a tightly regulated process that is thought to be self‐regulated. Importantly, enzymes involved in sphingolipid metabolism have been identified as targets for the *Runx* family of genes with glucosylceramide and ganglioside synthesis being upregulated and S1P catabolism downregulated upon *Runx* activation.^93^ Leukaemic cells take advantage of this by favoring S1P synthesis over ceramide production as ceramides induce apoptosis.^94^ S1P fosters leukaemic cell growth and survival by preventing apoptosis by inhibiting cytochrome c and Smac/DIABLO release from the mitochondria, ultimately preventing the terminal activation of caspase 3 (Figure 4).^95^ Accordingly, altering the metabolism by inducing ceramide synthesis or inhibiting S1P synthesis sensitises HL‐60 and U937 cells and in primary myeloblasts to irradiation and apoptosis.^96^, ^97^, ^98^ This does not appear to be limited to S1P metabolism, but rather to ceramide availability as chemotherapy‐resistant tumors have increased sphingomyelin and glucosylceramide synthesis compared to chemotherapy‐sensitive ones, concomitant with a decrease in ceramide‐induced apoptosis.^99^

Haematopoietic stem and progenitor cells with defective cholesterol efflux in a hypercholesterolaemic environment are noted to phenocopy myeloproliferative neoplasms (MPN), and, as such, cholesterol transport and synthesis are upregulated in certain leukaemias and lymphomas.^56^, ^100^ We have also shown that myelofibrosis and essential thrombocytosis, driven by an activating mutation in c‐MPL (c‐MPL^W515L^) found in human myeloproliferative neoplasms, are more aggressive when cholesterol efflux from cells is disrupted.^54^ c‐MPL is localised in lipid rafts to signal, and we found that promoting cholesterol efflux with reconstituted HDL decreased platelet numbers in WT mice carrying the c‐MPL^W515L^ mutation. A similar finding was also reported in JAK2^V617F^‐mediated proliferation, where lipid raft disruption decreased JAK2 signalling.^101^

Interestingly, LDLr activity is dramatically increased in people with acute myeloid leukaemia (AML; Figure 4), where the catabolic rate of plasma LDL is threefold higher than healthy controls suggesting a requirement of lipids in AML cells.^102^ Cholesterol synthesis through the mevalonate pathway is also important in AML, with inhibition of the rate‐limiting enzyme HMG‐CoA reductase with statins acting in a toxic manner in AML cells.^101^, ^103^ Interestingly, some AML cells appear to bolster their cholesterol levels (uptake and synthesis) in response to chemotherapy allowing them to survive.^104^ Again, targeting these pathways sensitises AML pathways to therapy.^104^ Sadly, even after successful ablation, AML relapse still remains a major issue and occurs through leukaemic stem cells (LSCs).^105^ However, targeting LSCs is challenging as it often comes at the expense of HSPCs as they are highly similar. Inhibiting cholesterol synthesis may also be effective in LSCs as statin treatments have been shown to decrease LSC stemness but not HSPCs.^106^

As a consequence of the interconnected nature of the various lipid metabolic pathways, lipid metabolites themselves exist in a coregulated network; for example, perturbations in sphingolipid metabolism induce knock‐on effects on various phospholipid species (Figure 4). This tightly regulated network can also affect leukaemic stemness. Taz inhibition induces phospholipid remodelling by decreasing cardiolipin levels in leukaemic stem cells and, consequently, increasing the abundance of PS.^107^ Of note, this remodelling did not affect mitochondrial integrity, but rather increasing PS abundance promoted Toll‐like receptor (TLR) activity, thereby inducing a shift from stemness to differentiation. Further phospholipid remodelling has been observed in various cancer cell lines, including leukaemia, demonstrated by an increase in ether lipid content, implicating them in carcinogenesis.^84^, ^108^ Interestingly, plasmalogen content in leukaemic cells determines sensitivity to synthetic lipid‐induced cytotoxicity with the HL‐60 cell line being more susceptible than K562 cells as they have higher plasmalogen content.^108^ Supplementing K562 cells with the plasmalogen precursor alkylglycerol increased plasmalogen content consequently made them more susceptible to chemotherapy,^108^ indicating that modulating the lipidomes of cancer cells has therapeutic potential.

The lipid metabolic phenotype observed in haematological malignancies has been described previously; however, with the knowledge that leukaemic stem cells have a vastly different lipidome to HSCs, future studies will need to focus on understanding how the lipidome differs in greater detail, providing more information into their lipid metabolism and potentially taking advantage of this to develop therapeutic targets.

## Future directions

Our understanding on the interactions between lipid metabolism and haematopoiesis is slowly evolving, where it is now well established that FFAs are used as an energy source for HSC self‐renewal and progenitor cell differentiation. However, lipid metabolism is an intricate network of different metabolic pathways that can be directly influenced by one another.^16^ From the findings discussed, lipid metabolism influences haematopoiesis in both a cell‐autonomous manner and through the BM microenvironment. Understanding how these different pathways are altered during haematopoiesis opens up new avenues in the metabolic regulation of haematopoiesis.

It is well established that the transcriptome of the HSPC population changes course during development with more lineage‐specific progenitor cells exhibiting quite distinct profiles at the transcript level. Extrapolating these findings onto the proteome as well as the metabolome may help elucidate more of the intricacies of haematopoiesis. An advance by the Morrison group demonstrates that the metabolome differs during haematopoiesis with HSCs and MPPs having similar metabolomes, while committed progenitor populations exhibit rather distinct profiles, indicating specific metabolic preferences between the haematopoietic sub‐branches.^109^ However, because of the low abundance of these cells, only 60 positively charged metabolites could be detected. With the emergence of more sensitive assays in the future, that is advances in mass spectrometry and optimising ionisation efficiency for negatively charged metabolites, and perhaps pooling biological replicates to acquire more starting material, a wider breadth of the metabolome and, in particular, metabolites relevant to the mitochondria may be able to be quantified to provide further insight into the metabolome of HSCs and even LSCs. Knowing that the metabolome undergoes changes during haematopoiesis, it is highly likely that substrate preference (and the source of these substrates) changes during blood cell development.

Whether haematopoietic cells can directly produce these lipids remains an important question as findings reviewed herein indicate that lipids derived from BM stromal cells are necessary for regulating HSC differentiation. The BM niche is composed of a myriad of cell types, and how they regulate haematopoiesis has been intensively studied for decades. The hypoxia created by the BM niche supports the metabolic demands of HSCs to self‐renew, and as they migrate to another part of the BM, their metabolism changes and, thus, they begin to differentiate.^8^ BM adipocytes regulate haematopoiesis, particularly through the production of lipid mediators. However, BM adipocytes, while being integral to the niche, are only a small part of a whole BM environment.^110^ BM adipocytes may receive FAs from endothelial cells to store lipids and to replenish neutral lipid stores when they undergo lipolysis. It may be possible that HSCs and progenitor cells also receive FAs or other lipid moieties in a similar fashion.

Metabolic regulation is a crucial aspect of haematopoiesis. While FAO is an important regulator of cell fate, nonoxidative lipid metabolism influences this process in many different ways. Investigating the questions posed above will broaden our understanding of lipid metabolism during blood cell development and leukaemia.

## Conflict of interest

The authors declare no conflict of interest.
